# Research on the mechanism of cow milk protein dietary intervention in ameliorating systemic chronic inflammation in type 2 diabetes by disrupting the ROS-M1 macrophage axis

**DOI:** 10.3389/fnut.2026.1758163

**Published:** 2026-02-04

**Authors:** Fumei Zhang, Lin Bai, Heqiang Yang, Jianrong Yang, Zhuxin Sun, Jia Wei, Zilin Qiao, Yumei Wei, Fei Song, Xiaojing Tian, Xiaoxia Hu

**Affiliations:** 1 Key Laboratory of Biotechnology and Bioengineering of State Ethnic Affairs Commission, Biomedical Research Center, Northwest Minzu University, Lanzhou, China; 2 Department of Medicine, Northwest Minzu University, Lanzhou, China; 3 School of Life Sciences and Engineering, Northwest Minzu University, Lanzhou, China; 4 Shengyuan Nutritional Food Co., Ltd., Qingdao, China; 5 The Second Hospital and Clinical Medical School, Lanzhou University, Lanzhou, China

**Keywords:** cow milk protein, macrophage polarization, oxidative stress, systemic chronic inflammatory response, type 2 diabetes mellitus

## Abstract

**Introduction:**

Recent studies suggest that type 2 diabetes mellitus (T2DM) is characterized by a systemic, low-grade chronic inflammatory state. Although cow milk protein (CMP) has been shown to alleviate this inflammation, its underlying mechanisms remain unclear.

**Methods:**

Therefore, we investigated how CMP mitigates systemic chronic inflammation in T2DM using both *in vitro* digestion and mouse models.

**Results:**

The *in vitro* digestion model demonstrated that CMP, with its low degree of hydrolysis, exhibits significant anti-α-amylase and antioxidant activities. In the *in vivo* study, CMP markedly reduced fasting blood glucose (FBG) and reversed diabetes-related body weight loss. CMP intervention significantly decreased oxidative stress markers, including malondialdehyde (MDA) and reactive oxygen species (ROS), while enhancing the activity of the antioxidant enzyme glutathione peroxidase (GSH-Px). Moreover, CMP suppressed macrophage polarization toward the M1 phenotype and reduced the levels of pro-inflammatory cytokines. Finally, CMP administration ameliorated lipid infiltration in the liver and intestine, mitigated pancreatic islet atrophy, and concurrently alleviated renal pathologies such as glomerular hypertrophy, glycation, and fibrosis.

**Discussion:**

In conclusion, CMP ameliorates systemic chronic inflammation in T2DM by disrupting the ROS–M1 macrophage vicious cycle.

## Graphical abstract

**Figure:**
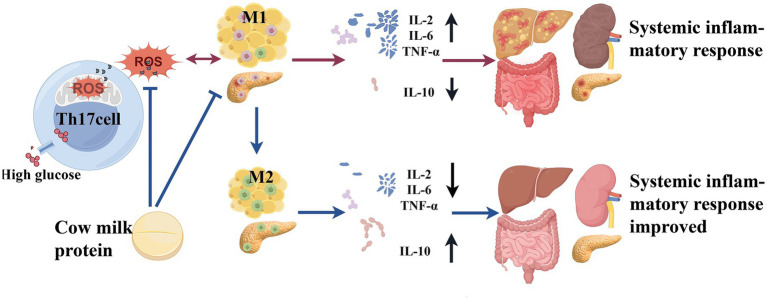

## 1 Introduction

According to the data released by the International Diabetes Federation in 2025, there are approximately 589 million adults with diabetes worldwide (1). This issue is particularly severe in China, where the number of patients has reached 233 million, representing a significant increase of 163.36% between 2005 and 2023 (2). Furthermore, the atypical clinical symptoms of diabetes and it’s unclear pathogenesis contribute to low awareness (36.7%), treatment (32.9%), and control rates (50.1%) (3), placing heavy burden on both patients and society. Therefore, based on the cost and benefit of treatment, the American Diabetes Association and the Academy of Nutrition and Dietetics recommend lifestyle changes, especially self-nutrition support therapy, to improve both the efficacy and the quality of life for individuals with pre-diabetes and T2DM (4, 5). It is well known that increasing dietary protein to 15–20% of total energy intake can effectively control blood sugar without adversely affecting renal function (6). Its effect is even comparable to some oral drugs (7). At the same time, protein induces a strong sense of satiety, and enhances insulin sensitivity and secretion (8). These properties make it a key component of functional foods for the prevention and adjuvant treatment of T2DM.

Numerous studies have demonstrated that oxidative stress and macrophage polarization play critical roles in chronic inflammation and insulin resistance associated with T2DM (9). Persistent hyperglycemia leads to excessive production of ROS through multiple pathways (10). Moreover, ROS not only directly activate immune cells as inflammatory signals but also stimulate macrophages to polarize into the M1 phenotype. This results in the secretion of large amounts of inflammatory cytokines such as TNF-α, IL-6, and IL-1β, which induces chronic inflammation in target organs, including adipose tissue and the liver, thereby contributing to insulin resistance (11). These pathological processes are interconnected, forming a vicious cycle of mutual reinforcement. Increased insulin resistance and β-cell damage exacerbate dysregulation of blood glucose and lipids, which in turn promotes further oxidative stress. This self-sustaining cycle amplifies the progression of diabetes and its complications. CMP, a high-quality and readily absorbed protein, exerts multiple beneficial effects. It helps control fasting blood glucose (FBG) (12), modulates insulin secretion and sensitivity (13), and regulates inflammatory responses via its antioxidant properties (14–16). However, whether CMP can modulate macrophage polarization remains unclear. Therefore, this study aimed to investigate the protective effects of CMP against systemic chronic inflammation in a T2DM mouse model. We focused specifically on its antioxidant properties and regulation of macrophage polarization.

## 2 Materials and methods

### 2.1 Materials and reagents

CMP was provided from Shengyuan Company (Shandong, China). Casein (CS) was purchased by Hualing Company (Gansu, China). Whey protein (WPC) was purchased from Glanbia Company (Kilkenny, Ireland). ELISA assay kits forinterleukin-6 (IL-6), interleukin-10 (IL-10), interleukin-2 (IL-2), and tumor necrosis factor-alpha (TNF-α) were purchased from Xinbosheng Biotechnology Co., Ltd. (Shenzhen, China). MDA, Superoxide Dismutase (SOD) and GSH-Px detection kits were purchased from Nanjing Jiancheng Bioengineering Institute (Nanjing, China). ROS detection kits were purchased from Shanghai Beibo Biotechnology Co., Ltd. (Shanghai, China). Pepsin (1:10000), trypsin (1:250), streptozotocin (STZ), Periodic acid-schiff (PAS) staining kit, hematoxylin–eosin (H&E) staining kit, Masson’s trichrome staining kit, and immunohistochemical primary antibodies (CD86, CD163) were purchased from Solarbio Technology Co., Ltd. (Beijing, China). Sitagliptin phosphate tablets (SIG) were purchased from Moshadong Pharmaceutical Co., Ltd. (Zhejiang, China).

### 2.2 *In vitro* semi-dynamic simulated digestion

Simulated gastric and intestinal fluids were prepared for *in vitro* digestion using a semi-dynamic simulated digestion model (17). To demonstrate the *in vitro* digestion characteristics of CMP, CS and WPC was used in different ratios for comparison. Therefore, a series of 2% milk protein solutions with varying ratios were prepared as detailed in Table 1. For the OPA assay, 30 μL of each sample was mixed with 240 μL of the OPA reagent. The absorbance was then measured at 340 nm (OD_340_) using a Multiskan™ FC microplate reader (Thermo, Massachusetts, United States) (18). A standard curve was generated using serine, and the degree of hydrolysis was calculated according to Equation 1.

(1) $$\text{Degree of hydrolysis}\;(\%)=(\text{Serine}\;NH_{2}-\beta )/(\alpha *h_{\mathrm{tot}})*100$$

**Table 1:** Milk protein grouping.

Group	Ingredients
I	100% CS
II	100% CMP
III	50% CS + 50% WPC
IV	20% CS + 80% WPC
V	100% WPC

In the formula, *Serine NH*_2_ is the content of serine amino groups per gram of protein, mmol/g. β and α are constants of milk protein. *h*_tot_ is the total number of milk protein peptide bonds.

### 2.3 *In vitro* evaluation of anti-α-amylase and antioxidant activities of digested products

The anti-α-amylase activity was determined by measuring the absorbance at 540 nm (OD_540_) using a Multiskan™ FC microplate reader (19). Two control groups were established, an experimental control without the starch solution and a sample control without the test sample. The α-amylase inhibition rate was then calculated according to Equation 2.

(2) $$\alpha -\text{Amylase inhibition}\;(\%)=(\begin{array}{l} 1-(\begin{array}{l} OD_{\text{sample}}-OD_{\text{sampleControl}}\\ -OD_{\text{blank}} \end{array})\\ /(OD_{\text{Control}}-OD_{\text{blank}}) \end{array})*100$$

In the formula, the *OD* value represents absorbance.

The hydroxyl free radical (·OH) scavenging ability was determined by measuring the absorbance of the reaction mixture at 536 nm (OD_536_) using a Multiskan™ FC microplate reader. The reaction mixture without any antioxidants served as the control group (20). Vitamin C (Vc) at a concentration of 0.1 mg/mL was used as the positive control, the ·OH scavenging ability of the samples was calculated according to Equation 3.

(3) $$OH_{\text{Clearance rate}}=(\begin{array}{l} 1-(OD_{\text{sample}}-OD_{\text{blank}})\\ /(OD_{\text{Control}}-OD_{\text{blank}}) \end{array})*100$$

In the formula, the *OD* value represents absorbance.

The total reducing power was assessed by the potassium ferricyanide reduction method, measured at 700 nm (OD_700_) using a Multiskan™ FC microplate reader. The reducing ability was expressed as the absorbance value. Vc at a concentration of 0.1 mg/mL was used as the positive control (21).

### 2.4 Animal experiment

**Figure 1:**
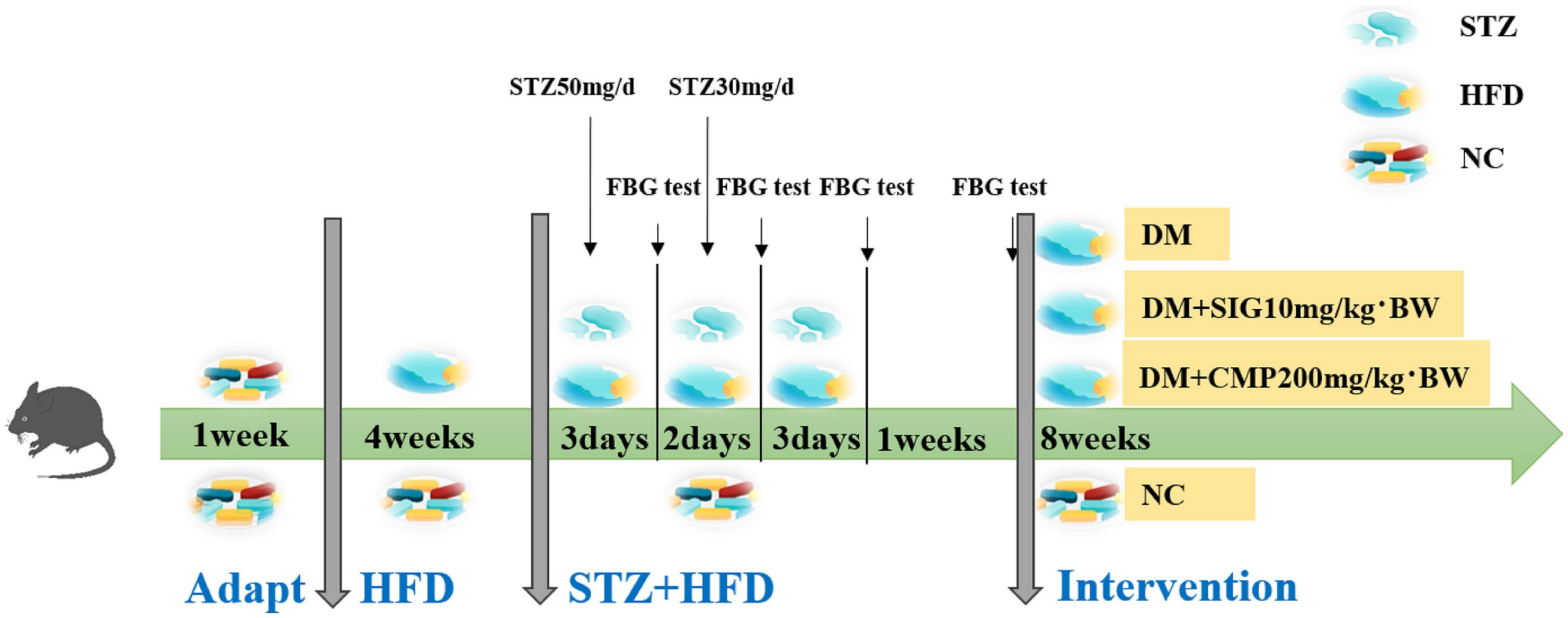
Schematic of the T2DM mouse model establishment and the subsequent dietary intervention process.

This study was approved by the Institutional Ethics Committee of Northwest Minzu University (ethics approval number: xbmu-sm-2024107). Male C57BL/6 J mice (6–8 weeks old, *N* = 48), weighing 22–25 g, were purchased from the Lanzhou Institute of Veterinary Medicine, Chinese Academy of Agricultural Sciences (Lanzhou, China). The animals were housed in a controlled environment with a constant temperature of 20 ± 5 °C and humidity of 50% ± 5%, maintained on a 12-h light/dark cycle, with free access to standard maintenance feed and pure water. After 1 week of acclimatization, the mice were randomly divided into two groups: a normal diet group (fat content 4.3% w/w, calorie content 10%, Lanzhou Veterinary Research Institute, Chinese Academy of Agricultural Sciences, Lanzhou, China) (NC group, *N* = 12) and a high-fat diet group (fat content 35% w/w, calorie content 60%, same source) (HFD group, *N* = 36). At the end of the fourth week, mice in the HFD group were intraperitoneally injected with STZ, dissolved in 0.05 M sterile sodium citrate buffer (pH 4.5), at a dose of 50 mg/kg for three consecutive days. FBG and random blood glucose levels were measured via caudal vein puncture using a handheld blood glucose meter. Mice with FBG ≤ 11.1 mmol/L received additional STZ injections at 30 mg/kg for 2 days. FBG was re-evaluated 3 days and 1 week after the final injection. Mice with FBG > 11.1 mmol/L at both time points were considered successfully modeled for T2DM. The normal control group received intraperitoneal injections of an equal volume of sodium citrate buffer concurrently (22). Following successful modeling, T2DM mice were randomly assigned to three groups (*N* = 12 per group): the type 2 diabetes mellitus model (DM) group, the SIG group, and the CMP group. Throughout the 8-week intervention, all mice received daily intragastric administration. The NC and DM groups were administered with pure water at a dose of 10 mg/kg/day, whereas the SIG and CMP groups received SIG (10 mg/kg/day) and CMP (200 mg/kg/day), respectively. Body weight and FBG levels were recorded every 2 weeks. The detailed experimental procedure is illustrated in Figure 1.

After 8 weeks of intervention, the animals were fasted for 12 h and anesthetized via intraperitoneal injection of 10% (w/v) pentobarbital sodium. Whole blood was collected from the inferior vena cava into 2 mL centrifuge tubes. The blood samples were allowed to stand at 25 °C for 2 h and then centrifuged at 247 × g for 15 min at 4 °C. The supernatant was transferred to 1.5 mL centrifuge tubes and stored at −80 °C. The mice were sacrificed by cervical dislocation, and the kidneys, liver, ileum, and pancreas were collected. The collected tissues were divided into two portions: one stored at −80 °C for future use, while the other was fixed in a fixative solution for histological analysis. Visceral adipose tissue was collected for flow cytometric analysis.

### 2.5 Biochemical index detection

FBG was measured via tail vein puncture using a blood glucose meter (GLM-77, YASEE Biotech, Qingdao, China). The levels of IL-6, IL-10, IL-2, and TNF-α in serum were detected with ELISA kits. Serum liver function markers [aspartate aminotransferase (AST), alanine aminotransferase (ALT)] and renal function indicators [creatinine (CREA), urea nitrogen (UREA), uric acid (UA)] were measured using a fully automated biochemical analyzer (SAL9000, Mindray, Shenzhen, China). Oxidative stress-related markers in liver homogenates were measured separately according to the MDA, SOD, GSH-Px, and ROS assay kits.

### 2.6 Flow cytometry analysis

The visceral adipose tissue of mice was digested using a mixed enzyme solution, and M1 and M2 macrophages were identified by flow cytometry. M1 macrophages were labeled with CD86^+^, and M2 macrophages were labeled with CD163^+^; these cells were the quantified using flow cytometry (BriCyte E6, Mindray, Shenzhen, China) (22).

### 2.7 Histopathological examination

The renal sections were stained with H&E, Masson’s trichrome, and PAS stains, and the pathological changes in renal tissue were observed under a light microscope (23). Liver, pancreas, and ileum sections were examined following H&E staining. Immunohistochemical staining was performed on pancreatic paraffin sections, which were incubated with primary antibodies against CD86 and CD163, respectively, to indicate M1 and M2 macrophage expression (24).

### 2.8 Statistical analysis

All data were analyzed in triplicate using SPSS software (IBM SPSS Statistics 27, IBM, New York, United States). Intergroup differences were evaluated by the Waller–Duncan *post hoc* test following one-way analysis of variance (ANOVA). Bars within the same panel labeled with different lowercase letters indicate statistically significant differences (*p* < 0.05). Data are presented as mean ± standard deviation. Tissue section analysis was performed using ImageJ for Windows 64-bit (USA National Institutes of Health, Maryland, United States). GraphPad Prism software (GraphPad Prism 9.5, GraphPad Inc., California, United States) was used for data visualization and statistical plotting. Pearson correlation coefficients were calculated to assess pairwise associations among variables. The absolute value of the correlation coefficient (|r|) was used to represent correlation strength, with larger |r| values indicating stronger associations. In the figure, edge width is proportional to |r| and is used solely to improve visualization readability.

## 3 Results

### 3.1 Evaluation of the anti-α-amylase and antioxidant activities of digestion products

**Figure 2:**
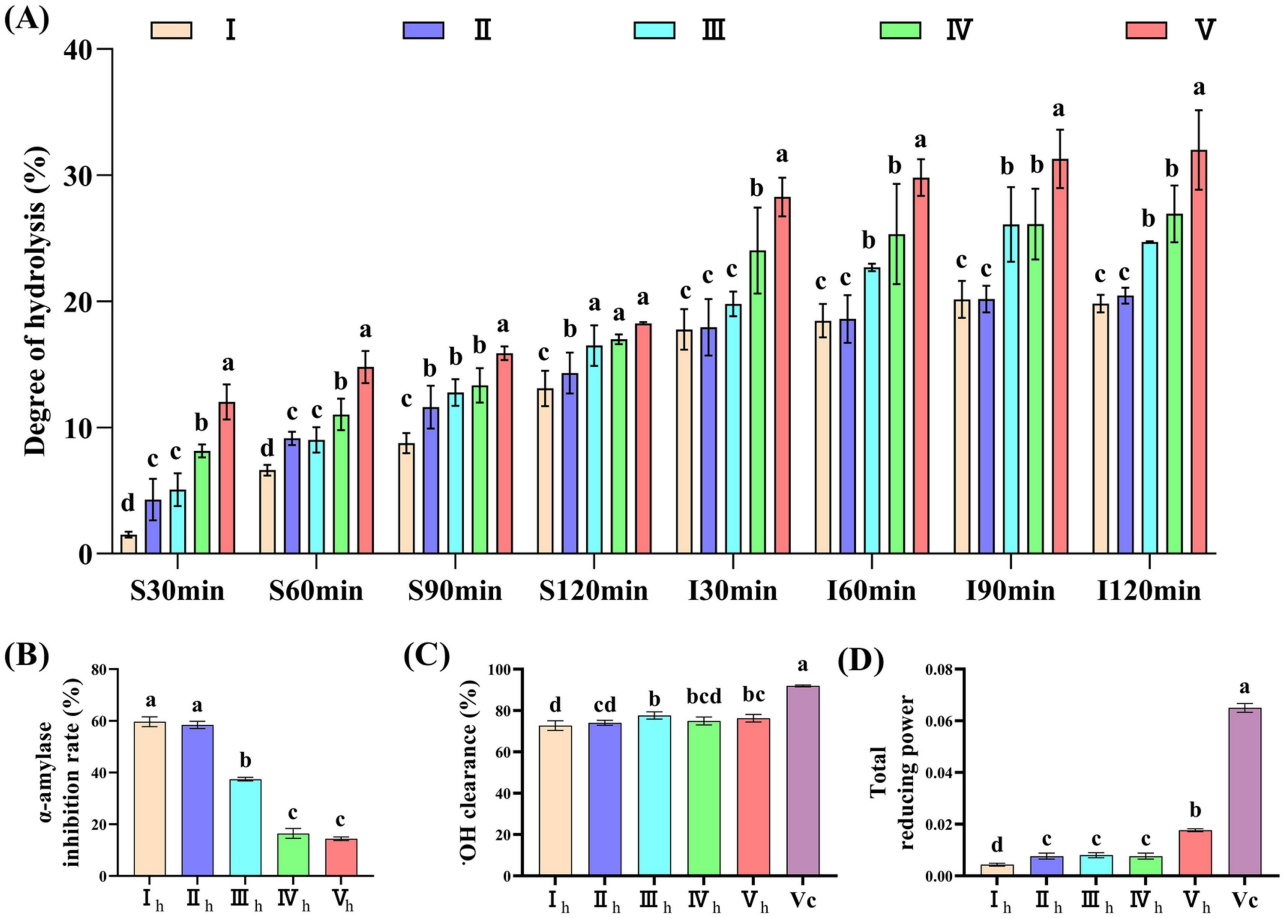
Comparative study on digestive characteristics and bioactivity of digestive products from five milk protein samples. **(A)** Digestive hydrolysis degree of five milk protein samples. S represents the gastric phase; I represents the intestinal phase. **(B,C)** I_h_: 100% CS hydrolysate; II_h_: 100% CMP hydrolysate; III_h_: 50% CS + 50% WPC hydrolysate; IV_h_: 20% CS + 80% WPC hydrolysate; V_h_: 100% WPC hydrolysate. **(B)** α-Amylase inhibitory activity. **(C)** ·OH free radical scavenging activity. **(D)** Total reducing power of the potassium ferricyanide reduction method. Bars labeled with different lowercase letters within the same panel are significantly different (*p* < 0.05) (The same applies to other figures).

A semi-dynamic digestion model was used to simulate the digestion of milk proteins, with the results presented in Figure 2. During the simulated gastric phase, the degree of hydrolysis of 100% CMP was significantly higher than that of the 100% CS groups at 30, 60, 90, and 120 min (Figure 2A, *P* < 0.05). Throughout the intestinal phase, the degree of hydrolysis of the 100% CS and 100% CMP groups was significantly lower than that of the 20% CS + 80% WPC and 100% WPC groups at all time points, while no significant difference was observed between the 100% CS and 100% CMP groups (Figure 2A, *P* < 0.05). The *in vitro* α-amylase inhibition rates of 100% CS and 100% CMP hydrolysate were significantly higher than those of the other groups (Figure 2B, *P* < 0.05). The ·OH free radical scavenging rate of 100% CMP hydrolysate was lower than that of 50% CS + 50% WPC hydrolysate, and there was no significant difference between 100% CMP hydrolysate and the other three groups (Figure 2C). The ferricyanide reducing power of the 100% CMP hydrolysate was significantly stronger than that of the 100% CS hydrolysate, but weaker than that of the 100% WPC hydrolysate (*p* < 0.05). There was no significant difference between the remaining two groups (Figure 2D). Therefore, CMP was selected for subsequent *in vivo* experiments due to its excellent digestibility, anti-α-amylase activity, and antioxidant properties.

### 3.2 Effects of CMP on fasting blood glucose and body weight

**Figure 3:**
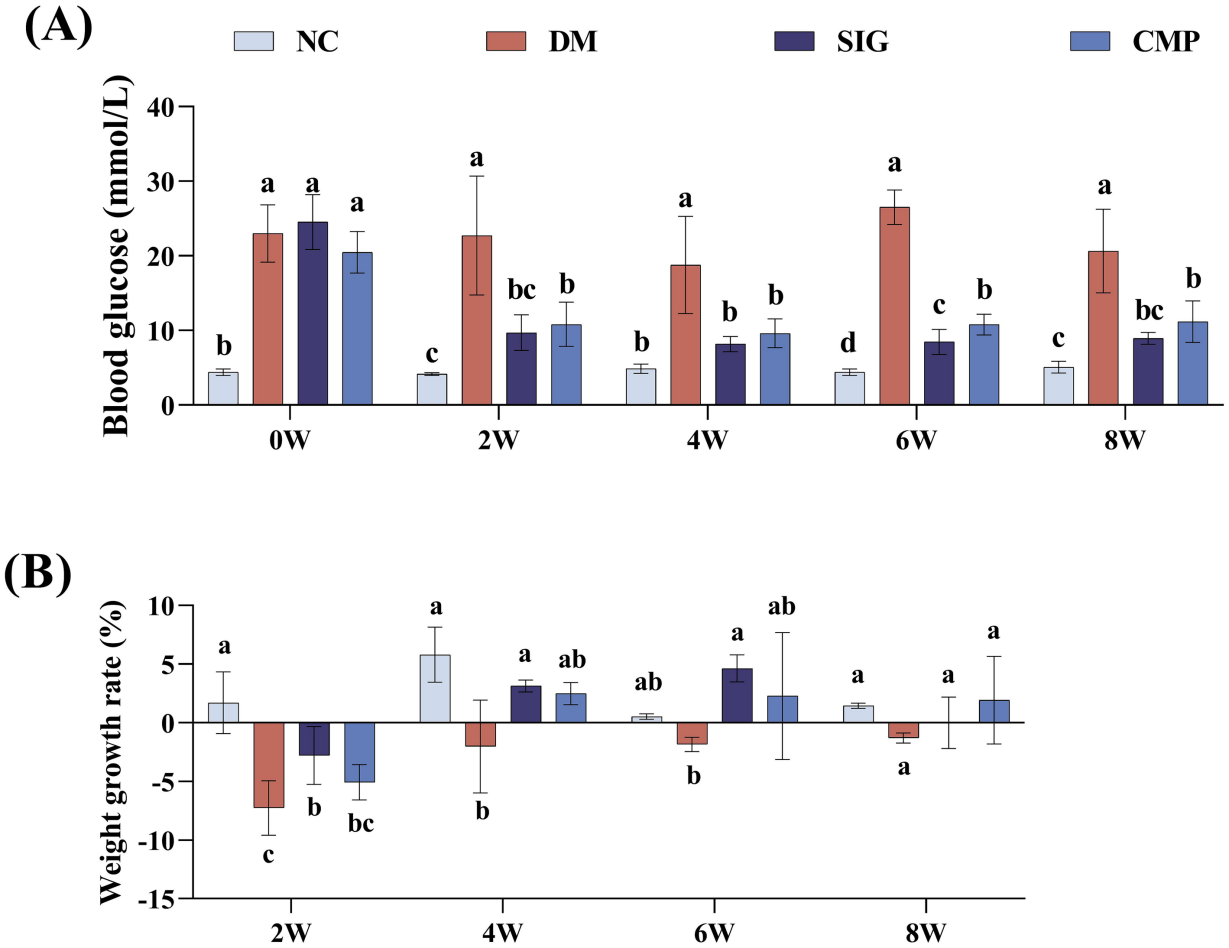
Effects of cow milk protein on blood glucose and body weight in T2DM mice. **(A)** Effect of CMP intervention on fasting blood glucose in T2DM mice. **(B)** Effect of CMP intervention on body weight gain rate in T2DM mice (the table showing the “mean ± standard deviation” of mouse weight gain is included in the Supplementary material).

To explore the effect of CMP on the basic physiological indices of T2DM mice, body weight and FBG levels were monitored throughout 8 weeks intervention period. The results were presented in Figure 3. FBG levels in the NC group stayed within the normal range for the entire intervention period. At the beginning of the intervention (week 0), FBG levels in the DM, SIG, and CMP groups all exceeded 11.1 mmol/L. The DM group remained hyperglycemic throughout the 8-week period. From weeks 2 to 8, FBG levels in both the SIG and CMP groups were significantly lower than in the DM group (*p* < 0.05), with no difference between them (Figure 3A). Body weight monitoring showed that the NC group exhibited continuous weight gain throughout the intervention, whereas the DM group showed sustained weight loss. CMP and SIG interventions gradually reversed this weight loss within 4 weeks, resulting in no difference compared to the NC group. At 6 weeks, the SIG group exhibited the highest weight gain rate. By 8 weeks, weight changes in all groups slowed, and body weight tended to stabilize (Figure 3B).

### 3.3 Effects of CMP on oxidative stress and macrophage polarization in T2DM mice

**Figure 4:**
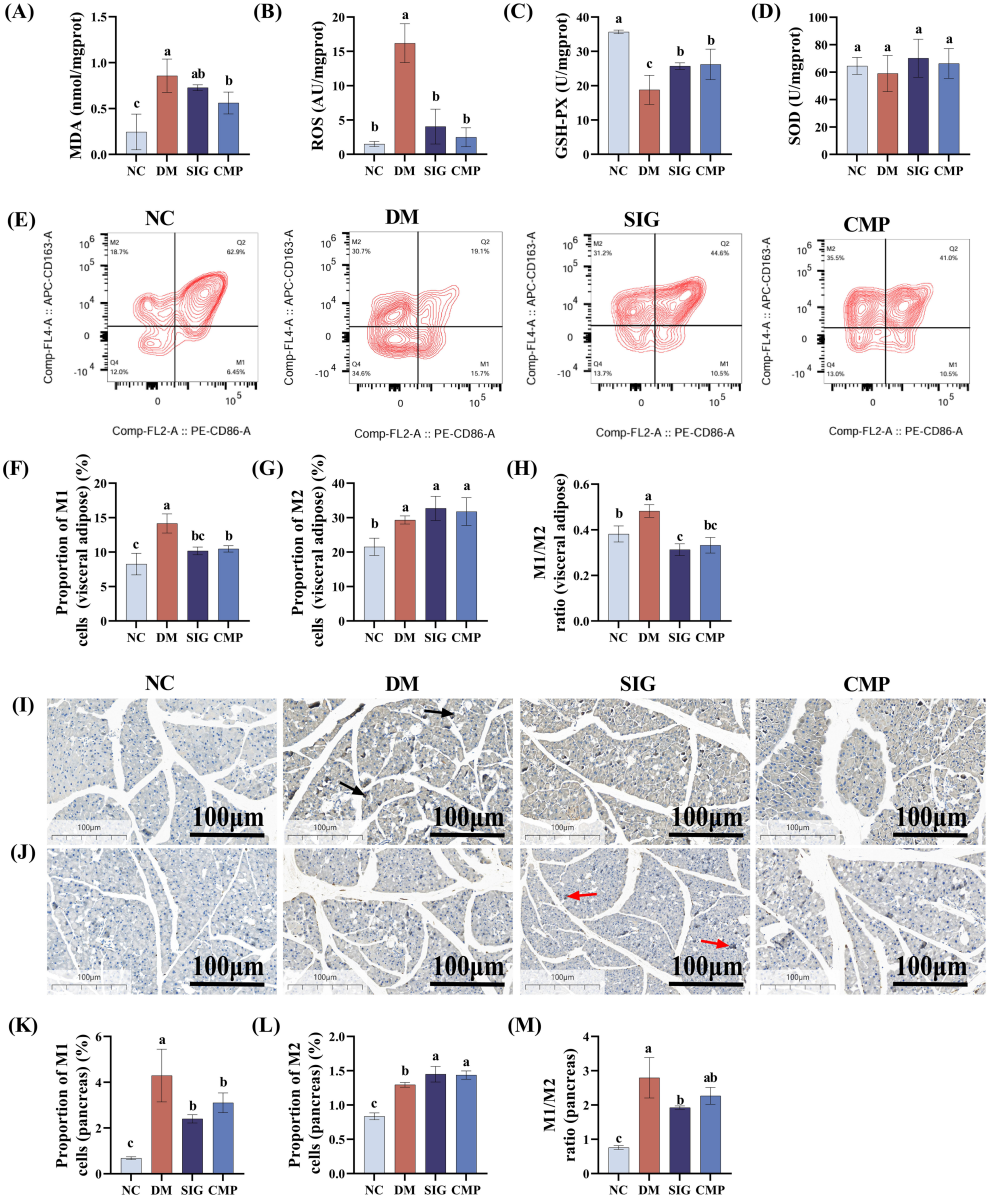
Effect of CMP on oxidative stress and macrophage polarization in T2DM mice. **(A–D)** Effects of CMP on oxidative stress markers: **(A)** MDA level, **(B)** ROS level, **(C)** GSH-PX activity, **(D)** SOD activity. **(E–H)** Flow cytometric analysis of M1/M2 macrophages in visceral adipose tissue after CMP intervention: **(E)** Representative flow cytometry plots, **(F)** percentage of M1-polarized macrophages (CD86^+^), **(G)** percentage of M2-polarized macrophages (CD163^+^), **(H)** M1/M2 ratio. **(I–L)** Immunohistochemical analysis of macrophage polarization in pancreatic tissue: **(I)** Expression of the M1 macrophage marker CD86, black arrows indicate positive cells, **(J)** expression of the M2 macrophage marker CD163, red arrows indicate positive cells, **(K)** percentage of M1-polarized macrophages, **(L)** percentage of M2-polarized macrophages. **(M)** M1/M2 ratio.

To explore whether CMP improves oxidative stress and macrophage polarization in T2DM, oxidative stress-related indicators and macrophage subtypes in visceral adipose tissue and pancreatic tissue were examined following CMP intervention, with the results presented in Figure 4. Compared with the NC group, the levels of oxidative stress markers MDA and ROS in the DM group were significantly increased (Figures 4A,B, *P* < 0.05). In contrast, the activity of the antioxidant enzyme GSH-Px was significantly decreased (Figure 4C, *P* < 0.05), while SOD activity was only slightly decreased (Figure 4D, *P* > 0.05). Compared with the DM group, both CMP and the positive control SIG groups restored the oxidative stress-related indicators to levels comparable with the NC group (Figures 4A–D). Meanwhile, compared with the NC group, the DM group exhibited increased expression of both M1 and M2 macrophages in visceral adipose and pancreatic tissue (Figures 4E–L, *P* < 0.05). However, the increase in M1 macrophages is greater than that in M2 macrophages, resulting in an elevated M1/M2 ratio at this time. Compared with the DM group, both CMP and SIG showed a similar regulatory trend. Both treatments reduced M1 macrophages expression (Figures 4F,K, *P* < 0.05) and increased M2 macrophages expression (Figure 4G, *P* > 0.05; L, *p* < 0.05). Critically, both interventions effectively lowered the M1/M2 ratio (Figures 4H,M, *P* < 0.05).

### 3.4 Effect of CMP on systemic chronic inflammatory injury in T2DM mice

**Figure 5:**
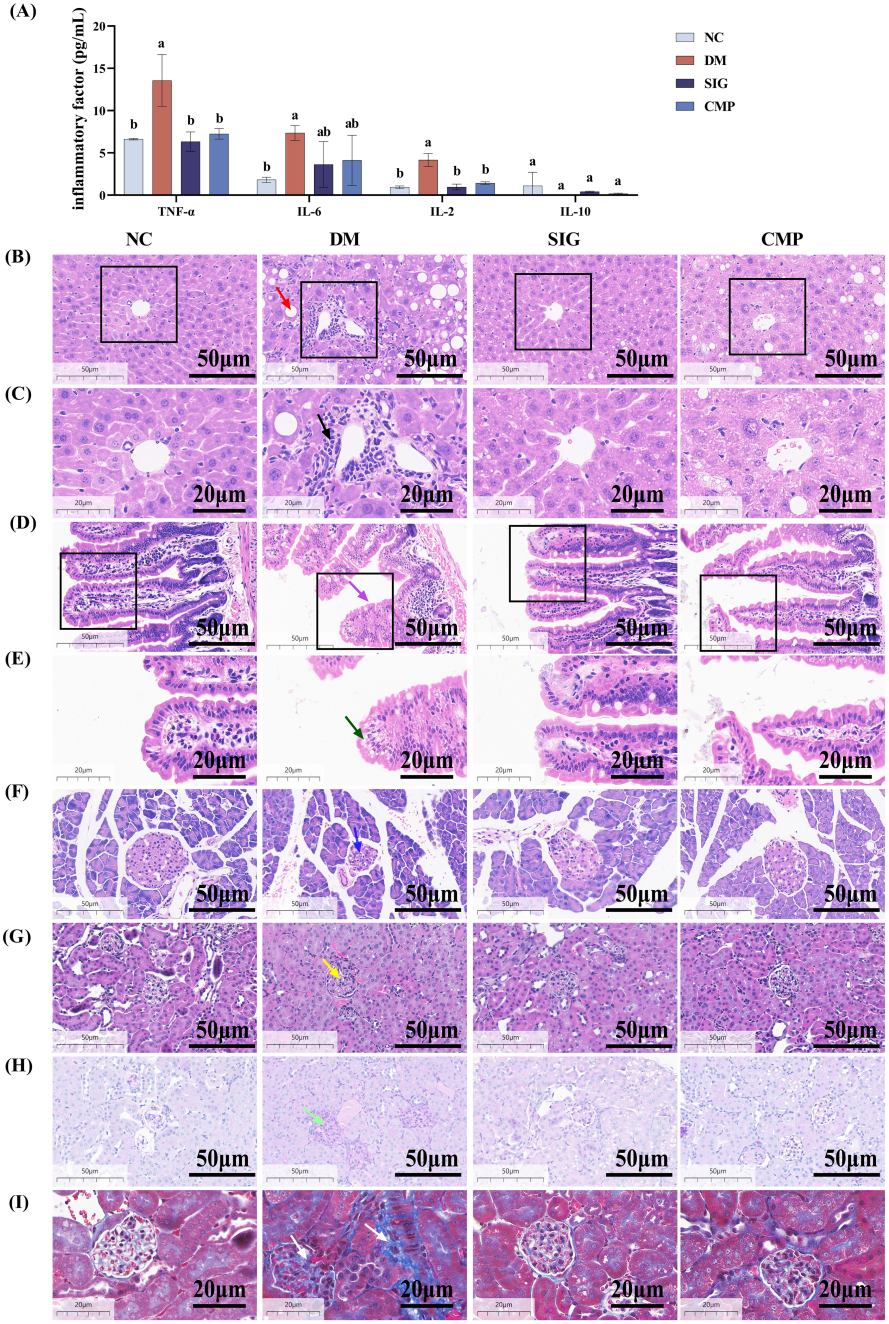
Effect of CMP on systemic inflammatory response in T2DM mice. **(A)** Effect of CMP on serum IL-2, IL-6, TNF-α, and IL-10 levels in T2DM mice. **(B)** Liver, HE staining, 40×, red arrow indicates lipid droplet vacuoles. **(C)** Liver, HE staining, 80×, black arrow indicates inflammatory infiltration. **(D)** Ileum, HE staining, 40×, purple arrow indicates intestinal villi. **(E)** Ileum, HE staining, 80×, green arrow indicates vacuoles at the tip of intestinal villi. **(F)** Pancreas, HE staining, 40×, blue arrow indicates islets. **(G)** Kidney, HE staining, 40×, yellow arrow indicates glomerulus. **(H)** Kidney, PAS staining, 40×, light green arrow indicates PAS-positive areas. **(I)** Kidney, Masson’s trichrome staining, 80×, white arrow indicates Masson-positive areas.

**Figure 6:**
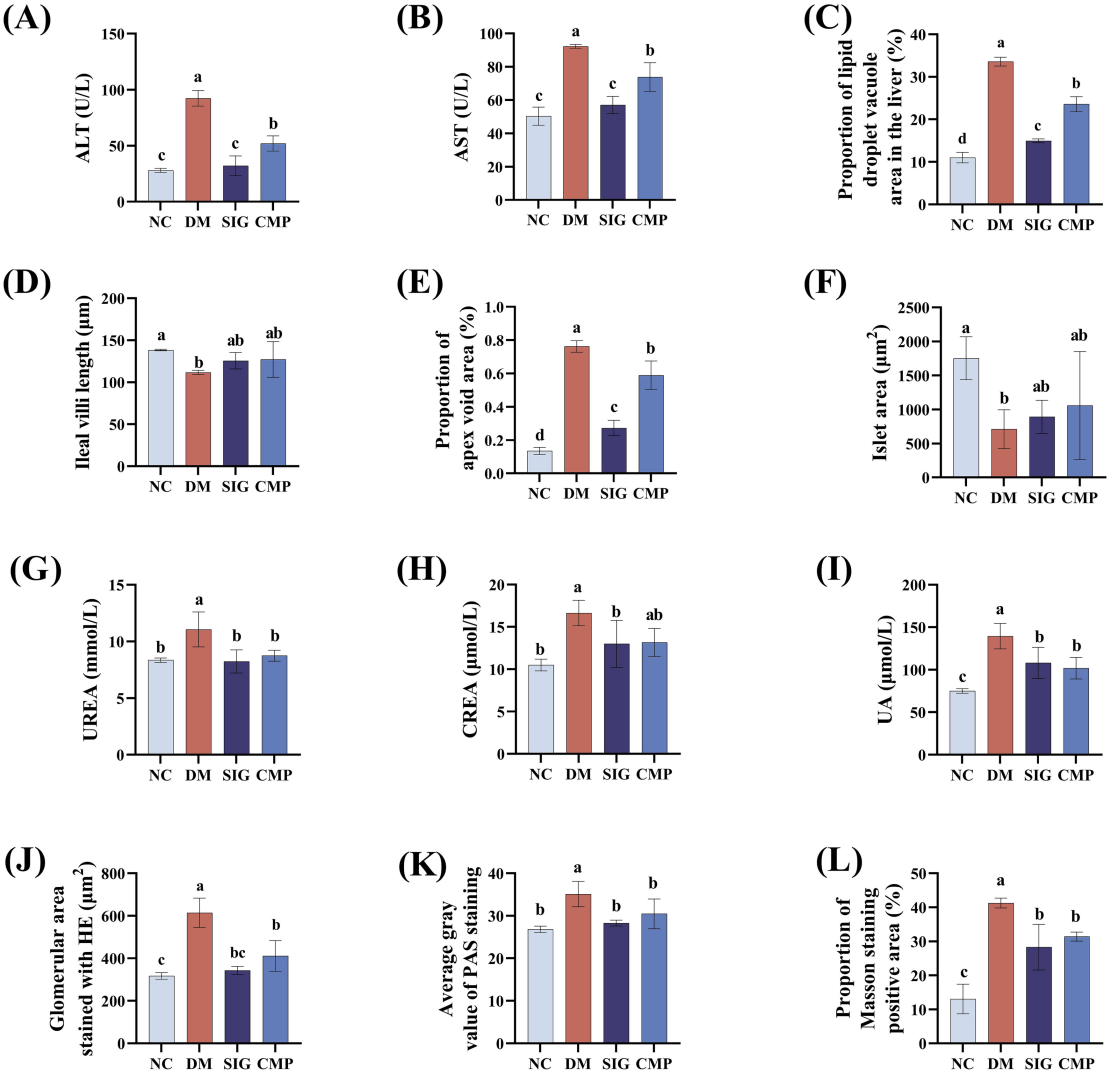
Liver and kidney function indicators and histopathological analysis. **(A)** Liver function: ALT activity. **(B)** Liver function: AST activity. **(C)** Hepatic adipose infiltration area. **(D)** Length of intestinal villi. **(E)** Area of adipose infiltration at the top of intestinal villi. **(F)** Islet area. **(G)** Renal function: Urea. **(H)** Renal function: CREA. **(I)** Renal function: UA. **(J)** Glomerular size. **(K)** Glomerular glycosylation. **(L)** Renal fibrosis.

We further investigated whether CMP alleviates systemic chronic inflammatory injury in T2DM mice by assessing serum inflammatory factors, liver and kidney function, and the histology of the liver, ileum, and pancreas. The results are presented in Figures 5, 6. Compared with the NC group, serum levels of the pro-inflammatory cytokines IL-2, IL-6, and TNF-α were markedly increased in the DM group (*p* < 0.05). CMP and SIG demonstrated a significant downward trend in IL-2 and TNF-α levels, achieving comparable effects with no significant difference from the NC group. IL-6 showed only a downward trend (Figure 5A). Serum biochemical analysis revealed that ALT and AST activities were significantly elevated in the DM group compared to the NC group. However, treatment with CMP and SIG resulted in a significant reduction of these enzyme levels (*p* < 0.05). And the effect was more pronounced in the SIG group than in the CMP group (Figures 6A,B). The DM group exhibited severe hepatic pathology, including disorganized cell arrangement, inflammatory infiltration, cellular swelling, marked steatosis, and narrowed sinusoids. Following intervention with CMP and SIG, these histopathological changes were visibly improved. Notably, the marked steatosis was significantly alleviated, as confirmed by quantitative analysis (Figures 5B, 6C, *P* < 0.05). The ileal villi in the DM group were markedly shortened, with a significant increase in adipose vacuolar area at the villus tips. These ileal injuries were significantly ameliorated after CMP and SIG treatments (Figures 5D,E, 6D,E, *P* < 0.05). The NC group exhibited islets with normal morphology, characterized by clear boundaries, regular shape, neatly arranged cells, deeply stained nuclei, and abundant cytoplasm. In contrast, the DM group displayed significant islet damage, including blurred boundaries, atrophy, cellular disarray, enlarged nuclei, and mild heterogeneity. Treatment with CMP and SIG effectively restored islet morphology and increased their area compared with the DM group (Figures 5F, 6F, *P* > 0.05). Serum levels of UREA, CREA, and UA were significantly elevated in the DM group compared to the NC group. However, intervention with CMP and SIG led to a significant reduction in these markers (Figures 6G–I, *P* < 0.05), although the reduction in CREA within the CMP group was not statistically significant. Mice in the DM group exhibited severe renal pathology, characterized by loosely arranged tissue, a disordered structure, marked mesangial expansion, and significantly enlarged glomeruli. However, these pathological alterations were markedly improved following intervention with CMP and SIG (Figures 5G, 6J, *P* < 0.05). The DM group exhibited significant thickening of the glomerular basement membrane, mesangial matrix expansion, and a pronounced increase in the deposition of PAS-positive substances along the glomerular capillary wall. Following CMP and SIG interventions, these pathological features were significantly ameliorated (Figures 5H, 6K, *P* < 0.05). Moreover, compared with the NC group, the deposition of blue collagen fibers in the glomeruli and renal tubulointerstitial was markedly increased in the DM group, while CMP and SIG treatments significantly improved this fibrotic change (Figures 5I, 6L, *P* < 0.05).

### 3.5 Correlation analysis

**Figure 7:**
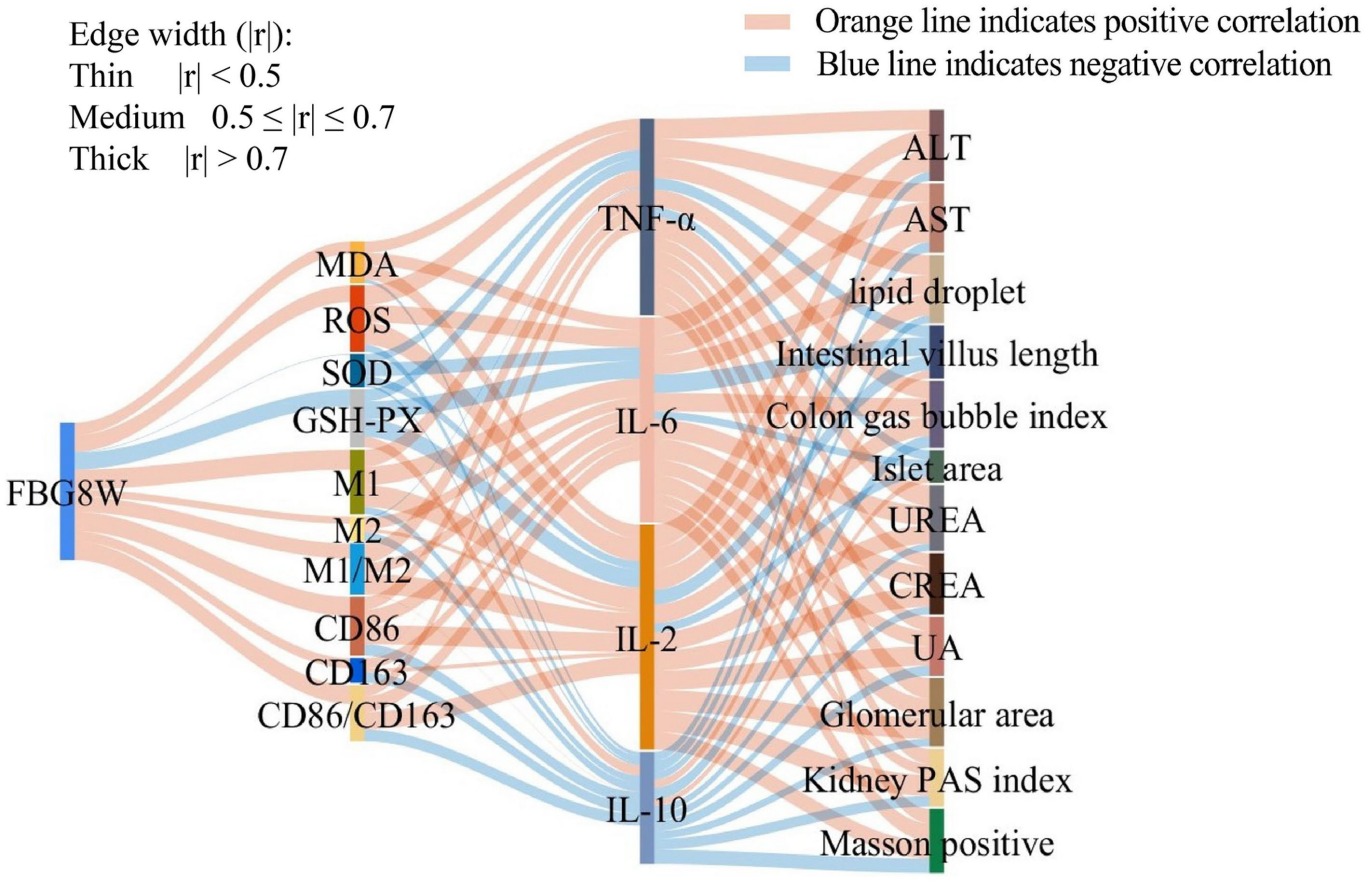
Correlation analysis of fasting blood glucose, oxidative stress, macrophage polarization indices, inflammatory cytokines, and systemic inflammatory response indicators in mice. Correlation analysis was performed using Pearson’s correlation test. Orange lines indicate positive correlations, while blue lines indicate negative correlations. Line width is proportional to the absolute value of the Pearson correlation coefficient (|r|), with wider lines indicating stronger correlations. Correlation strength was classified as strong (|r| > 0.7), moderate (0.5 ≤ |r| ≤ 0.7), or weak (|r| < 0.5).

Pearson correlation analysis was conducted to examine the relationships among FBG, levels of oxidative stress markers, markers of macrophage polarization, expression of inflammatory cytokine, and indicators of systemic chronic inflammation (Figure 7). FBG levels were positively correlated with oxidative stress factors (MDA, ROS), M1 macrophage levels, and the M1/M2 ratio, while negatively correlated with the activities of antioxidant enzymes (GSH-Px, SOD). Oxidative stress factors were positively correlated with pro-inflammatory cytokines (IL-2, IL-6, TNF-α), whereas antioxidant parameters were positively correlated with the anti-inflammatory cytokine IL-10. Pro-inflammatory cytokines showed positive correlations with indicators of liver and kidney function damage, including ALT, AST, CREA, UREA, and UA. They were also positively correlated with histopathological markers of damage, such as hepatic adipose infiltration, glomerular area, renal glycosylation, and fibrosis. Conversely, anti-inflammatory factor of IL-10 was positively correlated with pancreatic islet area and intestinal villus length.

## 4 Discussion

The main components of milk protein are CS and WPC. CS, which accounts for approximately 80% of milk protein, predominates during gastric digestion. It forms a clot in the stomach that increases the viscosity of gastric contents, thereby delaying gastric emptying and producing prolonged physical satiety. This process also leads to a moderate and delayed rise in plasma amino acid concentrations, therefore, CS is considered a “slow” protein (25). In contrast, WPC, a “fast” protein, can rapidly increase plasma amino acid levels to induce satiety and strongly promote insulin secretion (26), thereby rapidly reducing postprandial blood glucose. Although the effects of CS and WPC on satiety and food intake are not entirely consistent (27–30) both proteins effectively stimulate the gastrointestinal tract to release satiety hormones. By increasing plasma amino acid levels, they trigger the secretion of glucagon-like peptide-1 (GLP-1) (31, 32) and peptide YY (PYY) (33) which generate a strong signal to the brain to suppress appetite. In our study, *in vitro* semi-dynamic simulated digestion experiments demonstrated that CMP exhibited digestion kinetics similar to CS over the four-hour digestion period, with greater resistance to digestion compared to WPC. This property enables a stable and continuous supply of amino acids, thereby supporting prolonged satiety between meals. Furthermore, the supernatants of CMP and CS digests exhibited stronger anti-α-amylase activity than milk protein formulations containing higher proportions of WPC, whereas higher WPC content displayed superior antioxidant activity compared with CS digests. Therefore, considering these factors and the production input–output ratio, CMP was selected as the research subject in this study to further explore its potential mechanisms in the prevention and treatment of T2DM.

The core characteristics of T2DM include persistent hyperglycemia resulting from disordered glucose metabolism, clinically manifests as the classic symptoms of polydipsia, polyphagia, polyuria, and unexplained weight loss (34, 35). This study demonstrates that CMP effectively reduces fasting blood glucose levels in T2DM model mice, alleviates diabetes-induced weight loss, and exhibits therapeutic efficacy comparable to SIG treatment. We hypothesize that while CMP intervention suppresses appetite, it simultaneously provides substantial high-quality protein to the body. Furthermore, it may ameliorate insulin resistance and attenuate β-cell damage by reducing oxidative stress and modulating macrophage polarization.

Oxidative stress caused by the imbalance between ROS and the body’s antioxidant defense system is a key factor in the pathogenesis of T2DM and its related complications (36–38). Persistent hyperglycemia leads to excessive glucose metabolism in the mitochondria, generating large amounts of ROS such as superoxide anions, which are further exacerbated by the polyol and hexosamine pathways and the formation of advanced glycation end products (AGEs) (10). A decrease in glutathione levels reflects worsening oxidative stress (39). CMP intervention not only effectively reduced ROS and MDA levels in T2DM mice but also significantly increased the activity of the antioxidant enzyme GSH-Px. These findings indicate that CMP can target oxidative stress regulation and may provide a novel therapeutic option for managing T2DM and its complications. Meanwhile, ROS can induce the accumulation and activation of macrophages, leading to increased macrophage infiltration in adipose tissue, pancreas, and liver (40, 41). This process promotes a shift from the M2 to M1 phenotype and the secretion of inflammatory cytokines (TNF-α and IL-6), ultimately driving insulin resistance. Thus, regulating macrophage polarization represents a potential therapeutic strategy for T2DM (42). The results of this study indicate that both CMP and SIG effectively reduce the polarization of macrophages toward the M1 phenotype and promote their polarization toward the M2 phenotype, both in visceral fat and pancreatic tissue. More importantly, many scholars believe that reducing the M1/M2 ratio of macrophages is a more effective strategy for controlling systemic inflammation (43, 44). Following CMP intervention, the M1/M2 ratio in visceral fat and pancreatic tissue of T2DM mice was significantly reduced, consistent with the effects of SIG, indicating that CMP might act as a systemic immunoregulator to ameliorate T2DM.

Oxidative stress and macrophage polarization are not isolated events in the development of T2DM but rather form a vicious cycle of mutual promotion. Oxidative stress drives M1 macrophage polarization, which in turn enhances oxidative stress. This vicious cycle promotes the release of large amounts of inflammatory factors, induces insulin resistance, and contributes to β-cell damage. Consequently, blood glucose becomes more difficult to control, further exacerbates hyperglycemia, and generates more ROS. Ultimately, this vicious cycle amplifies continuously, driving the progression of diabetes and its complications. This study further investigated whether CMP can improve systemic chronic inflammation in T2DM mice. The results showed that CMP significantly inhibited the overexpression of pro-inflammatory factors (IL-2, IL-6, TNF-α) and promoted the expression of the anti-inflammatory factor IL-10. This is consistent with previous studies reporting that whey protein supplementation promotes wound healing in diabetic mice (45) and reduces the risk of familial aggregation in diabetic offspring (46). In T2DM mice, ALT and AST levels were significantly elevated, accompanied by increased liver steatosis and inflammatory infiltration. CMP supplementation improved liver function and effectively inhibited steatosis and inflammatory infiltration. The trend was similar to that observed with SIG, although the effect was weaker, indicating that CMP cannot replace pharmacological treatment but can serve as a nutritional supportive therapy. Consistent with the elevated inflammatory status, T2DM mice exhibited shortened intestinal villi, adipose infiltration at the villus apex, and atrophied islets. CMP administration mitigated these inflammation-associated tissue injuries, further confirming its anti-inflammatory efficacy. Diabetic kidney disease (DKD) is the most severe complication of diabetes (47). Given that targeting systemic chronic inflammation driven by oxidative stress (48, 49) and macrophage polarization (50, 51) is a critical strategy for managing DKD, our findings offer compelling evidence for CMP’s renoprotective effects. CMP intervention significantly restored renal function by reducing UREA, CREA, and UA levels, while simultaneously inhibiting structural damage such as glomerular hypertrophy and fibrosis in T2DM mice. Notably, these beneficial effects were achieved while providing high-quality protein, which aligns with the United States National Kidney Foundation’s clinical nutrition guidelines emphasizing the necessity of adequate protein intake to prevent malnutrition in chronic kidney disease (CKD) patients (52). Compared with red meat protein, milk protein may offer superior benefits in preventing and treating diabetes and improving renal hemodynamics (53, 54). Therefore, our study suggests that, under strict total protein intake control, milk protein can serve as a preferred source of high-quality protein to not only provide nutrition and improve metabolism but also deliver additional benefits through antioxidant effects, modulation of macrophage polarization, and anti-inflammatory actions.

This study aimed to further elucidate the underlying mechanism linking CMP intervention to the prevention and treatment of T2DM, focusing on its effects on oxidative stress, macrophage polarization, and systemic chronic inflammation. Pearson correlation analysis showed that, in T2DM, persistent hyperglycemia induced oxidative stress (elevated MDA and ROS levels) and M1 macrophage polarization, resulting in the release of large amounts of pro-inflammatory cytokines (IL-2, IL-6, TNF-α), which led to impaired liver and kidney function and multi-organ damage. Conversely, increased antioxidant enzyme activity (SOD, GSH-Px), M2 macrophage polarization, and a decreased M1/M2 ratio promoted the release of anti-inflammatory cytokines (IL-10) and inhibited systemic inflammatory responses. These findings provide a solid theoretical basis for CMP as a potential functional food for the prevention and adjuvant treatment of T2DM. Our work illuminates a novel therapeutic paradigm by targeting the interconnected pathways of oxidative stress and macrophage polarization. However, the optimal dosage, applicable population, and intervention duration of CMP still need further investigation.

## 5 Conclusion

This study confirms that CMP exhibits anti-α-amylase activity and antioxidant properties *in vitro*. *In vivo*, it can protect vital organs by regulating oxidative stress, improving macrophage polarization, and alleviating systemic chronic inflammatory responses. These findings provide crucial experimental evidence supporting CMP as a potential functional food for the prevention and adjunctive treatment of T2DM.

## Data Availability

The raw data supporting the conclusions of this article will be made available by the authors, without undue reservation.
