# Supplementary material for: Research on the mechanism of cow milk protein dietary intervention in ameliorating systemic chronic inflammation in type 2 diabetes by disrupting the ROS-M1 macrophage axis
Source: Front Nutr. 2026 Feb 4;13:1758163. doi: 10.3389/fnut.2026.1758163 (PMC12913101; doi:10.3389/fnut.2026.1758163)
Supplement: Supplementary file 1 [file Table_1.docx]

Supplementary Material

# Weight growth rate

|  | 2W (%) | 4W (%) | 6W (%) | 8W (%) |
| --- | --- | --- | --- | --- |
| NC | 1.69 ± 2.63^a^ | 5.79 ± 2.35^a^ | 0.53 ± 0.24^ab^ | 1.44 ± 0.22^a^ |
| DM | -7.27 ± 2.33^c^ | -2.03 ± 3.95^b^ | -1.85 ± 0.62^b^ | -1.31 ± 0.43^a^ |
| SIG | -2.80 ± 2.46^b^ | 3.13 ± 0.51^a^ | 4.63 ± 1.16^a^ | 0.00 ± 2.19^a^ |
| CMP | -5.08 ± 1.51^bc^ | 2.48 ± 0.94^ab^ | 2.28 ± 5.41^ab^ | 1.93 ± 3.74^a^ |
